# Does poverty increase COVID-19 in Africa? A cross-country analysis

**DOI:** 10.1186/s13561-022-00399-3

**Published:** 2022-10-10

**Authors:** Etayibtalnam Koudjom, Sévérin Tamwo, Koffi D. Kpognon

**Affiliations:** 1 Laboratory of Agricultural Economics and Applied Macroeconomics (LEAMA), University of Lomé, Lomé, Togo; 2 Center for Studies and Research in Economics and Management (CEREG), University of Yaoundé II-Soa, Yaoundé, Cameroon; 3 African Development Bank, Abidjan, Côte d’Ivoire, Institutional Economics Research Team (ERECI), University of Lomé, Lomé, Togo

**Keywords:** COVID-19, Poverty, Africa, I10, I32

## Abstract

**Background:**

Most economies in African countries are informal. As such, households in these countries tend to face higher levels of informality coupled with a lack of social protection, and have no replacement income or savings in the event of unexpected external shocks, such as COVID-19. Thus, the COVID-19 shock and its negative economic effects triggered a cascade of income losses and bankruptcies that pushed a significant share of households in African countries into poverty. This research analyzes the effect of poverty on the spread of COVID-19 using a sample of 52 African countries.

**Methods:**

To achieve the objective of this research, this paper uses a multiple linear regression model and a sample of 52 African countries observed in 2020 to conduct a cross-country analysis. More importantly, two COVID-19 indicators (total number of officially reported cases and disease severity) and six poverty indicators (average poverty, poverty incidence, poverty depth, poverty severity, multidimensional poverty index, and extreme poverty) were used in this research.

**Results:**

The results show a positive and significant relationship between poverty and the spread of COVID-19.

**Conclusions:**

These results suggest that more attention needs to be paid to poor populations in African countries during the pandemic. These populations are generally vulnerable, and there is a need for support programs targeting them to be put in place quickly. These programs may include food aid, distribution of supplies, health care support, fee waivers, and interest deferrals. In addition, sensitization of these disadvantaged groups on vaccination against COVID-19 to achieve herd immunity is strongly encouraged.

## Background

The Coronavirus disease 2019 (COVID-19) pandemic and containment measures have dealt a severe blow to the global economy [1–3]. Thus, a recession of -4.4% is expected, which is worse than the economic contraction observed during the global financial crisis of 2008–09 [4]. In sub-Saharan Africa (SSA), this global shock is expected to contract the economic activity by 2.8 percent in 2020 amidst high uncertainty, compared to 2.2 percent in 2019 [5]. According to the new projections, the pace of growth in emerging markets and developing countries from 2021 to 2023 will also not be sufficient to offset the output losses inflicted by the pandemic-related shocks [6]. Focusing on an annual basis, the Gross Domestic Product (GDP) of all developing countries is expected to remain below the pre-pandemic trend. According to the [7], other regions are faced with much larger output gaps relative to the pre-pandemic trend. In the specific case of SSA, the gap in 2023 relative to the pre-pandemic trend is expected to be more than 4%. Two theoretical streams attempt to explain the effects of COVID-19 within the framework of shock theory.

The first stream based on the Keynesian theory of aggregate supply and demand explains economic fluctuations driven by shocks due to their negative effects on aggregate supply and demand [8]. COVID-19 created a situation in which the supply and demand of goods and services temporarily stopped, bringing countries to the brink of economic recession [1]. To better understand the effects of the pandemic on aggregate supply and demand, it is important to look at the mechanisms by which the pandemic affects the economy. According to [9] and [10], there are three main channels through which the effects of the pandemic are transmitted. The first channel is the direct impact on reduced consumption of goods and services. Indeed, the prolonged duration of the pandemic and social distancing measures could reduce consumer confidence, who will become more wary of discretionary spending and pessimistic about long-term economic prospects [11]. The second is the indirect impact of financial market shocks on the real economy. Household wealth is likely to decline, as are household savings and consumption. The third is related to the disruptions that can occur on the supply side. Since COVID-19 keeps production low, it will have a negative effect on supply chains, labour demand, and employment, leading to extended periods of layoffs and increased unemployment. Moreover, the pandemic may create an anticipation shock from which a wait-and-see attitude from economic agents could be adopted by altering their transactions or consumption behaviors [12].

The second Stream highlights the effects of pandemics, armed conflicts or natural disasters on the economy. This theoretical trend globally shows that the macroeconomic effects of crises are the result of the contagion resulting from the spread of a health shock from one country to another. Their effects are felt in worldwide economies because the infection itself is widespread or because trade and market integration in capital and/or labor markets spread economic shocks across borders [13]. The negative impact of the pandemic is greatest for the most vulnerable segments of society. The pandemic has led to an economic downturn that could push millions of people into poverty. A rapid simulation including 138 developing and 26 high-income economies found that even in the most moderate scenario, COVID-19 could impoverish an additional 85 million people [14]. Similarly, [15] indicate that 49 million people could be pushed into extreme poverty in 2020 (living on less than $1.90 per day). These considerations are based on the fact that poor households have limited savings and food reserves. As a result, they can rarely work remotely and often rely on income from daily manual labor [2].

The poor living conditions of households in most African countries make them more vulnerable to shocks, including climate shocks, agricultural shocks and health shocks, etc. [16]. This vulnerability to shocks is attributable to constraints related to income growth, lack of employment, and risks associated with loss of land, assets, equipment, and infrastructure creating difficult conditions for these poor households to insure against these shocks [16, 17]. These devastating effects of shocks, especially health shocks on economic activity sustained by poverty amplify the spread of the shock and therefore prevent an exit from the crisis. Theoretically, poverty affects the transmission of health shocks due to the vulnerability of poor people [18]. To this end, it acts doubly in favor of the spread of a pandemic like Covid-19 through its joint effect on disease prevention and control.

With regard to prevention, in the event of a pandemic, poor households^1^ are unable to comply with the barrier measures enacted to stop the evolution of the disease, namely: the use of nose plugs^2^ and the practice of confinement. These households do not have enough money and are unable to obtain it or to remain confined to their homes, as they are heavily dependent on daily income from the informal sector. Failure to comply with these preventive measures due to their socioeconomic status is a catalyst for the spread of the disease [19, 20]. With regard to treatment itself, this segment of the population does not have sufficient financial means to access not only the health system, but also the quality health system [21, 22]. Non-compliance with preventive measures combined with lack of care limits the effectiveness of disease control, acting favorably to the spread of the disease. In addition, by remaining confined to their homes, they are at high risk of extreme poverty.

Other empirical tests confirm these results. [23] find an amplification of poverty in South Asia and SSA, with a greater effect in rural areas. Likewise, [24] shows that the number of people living on less than $1.90 per day could increase by 68 million in 2020 alone. Corroborating the previous results, [25] in the case of India, show that about 150 to 199 million additional people are expected to become poor in 2020. In the same vein, [26] show on a sample of 170 countries that the pandemic has had a significant effect on poverty increase. These results are also supported by the work of [27] in the case of the Bay Area (San Francisco). In the case of the West African Economic and Monetary Union (WAEMU), [28] show, using three scenarios of household income reduction (5%, 10% and 25%), that the incidence of poverty could increase in these countries. More recently, [29] in the case of Mozambique show that the pandemic increased poverty from 4.3 to 9.9%. [30] based on a sample of 3905 households, find overall that COVID- 19 had a negative effect on the standard of living in Ghana. [1] show from a qualitative survey that poverty had an effect on the spread of COVID-19 in Colombia.

Although these results are impressive, they focus on the impact of Covid-19 worldwide. Few studies have presented empirical verification of the factors that may explain the rapid spread of this pandemic worldwide. [3] demonstrated that the informal sector was the main cause of the spread of the pandemic in a sample of 46 sub-Saharan African countries. Similarly, [31] show through a sample of 182 countries that a population aged at least 65 years, population density and urbanization enhance the spread of the disease meanwhile the average temperature around the first quarter (January-March) acts in the opposite direction. However, bare studies have attempted to provide empirical evidence based on the populations’ standard of living. Authors who have attempted to analyze this issue have found conflicting results. Focusing on two poor neighborhoods in Ghana and South Africa, [19] find that the main factors hindering the effectiveness of containment policies are: poverty and lack of infrastructure. [20], in the case of South Africa, also note that containment is a major challenge for rural populations and those with more precarious livelihoods. [32], supports these ideas in the case of Chile by showing that containment was effective in containing and reducing new coronavirus cases in high-income municipalities. However, in low-income municipalities, it had a negative effect. [3] find no significant effect of health care quality and income on the disease spread. These contradictions in the prior literature prompt us to reexamine the relationship between poverty and the spread of Covid-19 in Africa.

There are several reasons explaining why Africa is considered an ideal area for such a study. Firstly, although in February 14, 2020, Africa accounted for only 3.5% of the 204.2 million laboratory-confirmed cases and 4.1% of the 4.3 million deaths reported worldwide [33], it has been heavily affected. This is because African countries have been affected differently by this disease, and the measures taken to stop the progression of the disease have sometimes differed from one country to another. On the bases of the year 2020, West Africa encountered 242,845 cases and 3247 deaths; North Africa registered 924,629 cases and 24,101 deaths. In Southern Africa, South Africa alone recorded 1,157,997 cases and 34,080 deaths. Eastern Africa recorded 322,826 cases and 6092 deaths, and Central Africa registered 70,234 cases and 1390 deaths. The higher number of cases and deaths in Southern Africa is imputed/attributed to South Africa, which was the most affected country on the continent during the same period. However, although Seychelles was the least affected country on the continent with 226 cases and 0 deaths, East Africa was not the least affected area [34]. This heterogeneity between subregions and/or countries creates health emergencies that force states to implement containment policies for the spread of the disease.

In terms of measures taken to stifle the spread of the disease, if some countries like South Africa and Uganda applied strict containment, others like Nigeria, Ghana and Tanzania applied less strict containment. moreover, in Ghana and Tanzania, the gathering of more than 25 and 10 people respectively was prohibited while in Sierra Leone, it took up to 101 people for a gathering to be banned [35]. Furthermore, according to a recent report by the [36], the continent’s budget deficit and public debt-to-GDP ratio has been significantly affected by the pandemic. The budget deficit reached an estimated peak of 8.1% of GDP in 2020. Meanwhile, over the same period, the ratio of public debt to GDP was above the 60% threshold that the [37] considers sustainable for African countries [36]. These upheavals are all the more striking given that the return to the pre-crisis situation is supposed to take place over several years.

Secondly, Africa is the poorest continent in the world. In recent years, the population in extreme poverty has reduced from 57% in 1990 to 43% in 2013 [38]. Despite this improvement, poverty outcomes remain far from the Sustainable Development Goals. According to the [39], the disruption caused by the COVID-19 pandemic led an estimated 55 million Africans into extreme poverty by 2020 and reversed more than two decades of progress in poverty reduction on the continent [39]. In this region, households facing hunger and relying on daily informal incomes may continue to go about their business [3, 40], putting themselves at greater risks of potential infections [34]. To stem the negative effects of the crisis on the poor and vulnerable, African governments have increased social assistance in the form of cash and in-kind transfers. By 2020, the cash and in-kind transfer base accounted for 74% of all social protection programs, well above the global average of 62%. Unfortunately, the average amount of social transfers was too small to increase consumption by the poor and lift them out of poverty. As a result, the prevalence of poverty in Africa has led to crowded markets, congested streets, and shared sanitation facilities, which can exacerbate/accelerate the spread of disease.

The objective of this research is therefore to fill this gap in the literature by analyzing the effect of poverty on the spread of COVID-19 in a sample of 52 African countries. This paper makes a threefold contribution to the existing literature. Firstly, it focuses on six indicators of poverty in order to expand empirical literature, which lacks consensus on the issue. Most of the work are interested on two or three indicators that may be irrelevant for assessing their effects on the spread of the disease. [2] focus on three indicators of poverty (the poverty ratio, a binary indicator, and a tertiary poverty indicator), while [23] focus on two indicators (the ratio of people living below the poverty line and a depth of poverty indicator). however, in addition to using a multidimensional poverty index that is more suitable for such a study [1], we use five different poverty indicators such as: average poverty, poverty incidence, poverty depth, poverty severity and extreme poverty. The multidimensional poverty index can be interpreted as an indicator of COVID-19 risk, as it assesses access to education, health, and housing [1].

In addition to these poverty indicators, we also use two COVID-19 indicators, including total number of cases and disease severity. Secondly, very few studies have empirically assessed the potential effect of poverty on the spread of the pandemic. To our knowledge, only the work of [1] evaluates this relationship in an exploratory manner in the case of Colombia. [2] simply showed that poverty affects labor mobility in four African and five Latin American countries. Meanwhile, [19] showed that poverty increases non-compliance with barrier measures. Thirdly, this study takes into account potential endogeneity issues that may bias our results and incorporates estimates for the years 2021 and 2022 as a robustness measure. Very few studies have provided empirical evidence on the year 2022. Overall, our results show that poverty improves the spread and severity of the disease. Section 2 presents the methods, Sect. 3 presents the results and discussion, and Sect. 4 concludes.

## Methods

### Model analysis

To analyze the relationship between COVID-19 spread and poverty, we rely on a multiple linear regression model developed by [3]. This model contains control variables such as population size and density, income levels as measured by GDP per capita, the quality of the health system as represented by public health expenditure as reported in the work of [41]. We also incorporate control variables such as official development assistance due to the importance of global governance in controlling the spread of disease [42, 43]. Institutional quality represented by corruption control and government effectiveness due to policy responses to the spread of the disease [44], and the Gini index to control for the level of income inequality. The rational for using this index is that in the literature, authors show that the difference in income between individuals has been the cause of non-compliance with barrier measures [2, 19]. Finally, to measure the spread of COVID-19, we consider the total number of officially confirmed cases from March 2020 to December 2020. For all that, this research considers disease severity^3^ to account for the severity of disease described by the frequency of clinical symptoms, complications of COVID-19, and outcome of COVID-19 infection [34], from March 2020 to December 2020. Thus, the empirical specification of the model takes the following form:

$${LogY}_{i}=\alpha +{\beta *Poverty}_{i}+{X}_{i}^{^{\prime}}*\gamma +{\varepsilon }_{i} (1)$$

where $Log{Y}_{i}$ is the Neperian Logarithm of the dependent variable which can be either the total number of confirmed COVID-19 cases or the COVID-19 severity in country $i$, ${Poverty}_{i}$ is our main variable of interest which can be one of the six poverty indicators namely: average poverty, poverty incidence, poverty depth, poverty severity, multidimensional poverty index, and extreme poverty in country $i$, ${X}_{i}$ is a $k\times 1$ vector of control variables consisting of: population size, population density, GDP per capita, official development assistance, health spending, corruption control, government effectiveness, and Gini index, and ${\varepsilon }_{i}$ the error term. β and $\gamma$ are coefficients to be estimated and $\alpha$ the constant. Thus presented, the model may suffer from endogeneity problems. Indeed, studies have shown that COVID-19 has an effect on poverty [28, 30]. Therefore, there is an endogeneity problem driven by reverse causality. To this end, to address these potential endogeneity problems, along with [3], we use pre-crisis poverty indicators. This excludes the possibility that these six poverty indicators are influenced by confirmed cases of COVID-19. The OLS method is applied for model estimation.

To test the robustness of our main results, we conduct two robustness tests using the same estimation technique. In the first test, we re-examine the relationship between poverty and the spread of COVID 19 over the year 2021 (January-December). In contrast, the second test looks at the year 2022 (January-June) focusing on the total number of cases, as data on the severity of the disease are not yet available in 2022.

### Data analysis

To achieve the objective of this research, this paper uses a sample of 52 African countries observed in 2020. The variables considered are those used in previous work (e.g., [3, 41–43]). Four data sources are used to collect the variables needed for the empirical analysis: (1) total number of confirmed COVID-19 cases and disease severity are from the [34]; (2) population size and density, official development assistance, GDP per capita, health expenditures are from World Bank indicators [5]; (3) the six poverty indicators, namely: average poverty, poverty incidence, poverty depth, poverty severity, multidimensional poverty index, extreme poverty, and Gini concentration index are taken from the World Bank PovcalNet Report [45]; (4) institutional variables, including control of corruption and government effectiveness are drawn from Worldwide Governance Indicators data [46]. Explicit definitions and data sources are summarized in Appendix Table 7. Moreover, there are also the appendices for a list of the 52 African countries selected for analysis arranged alphabetically. Descriptive statistics for the variables selected for analysis of the relationship between COVID-19 spread and poverty in Africa are presented in Table 1 below.

**Table 1:** Descriptive statistics of the variables selected for the analysis of the relationship between COVID-19 spread and poverty in Africa

Variables	Mean	Std. Dev	Min	Max
***Dependent variables***				
Total COVID-19 cases in 2020	52,431.940	155,166.000	226.000	1,039,161.000
Total COVID-19 cases in 2021	187,116.700	495,236.600	3877.000	3,446,532.000
Total COVID-19 cases in 2022	231,343.300	582,803.600	6043.000	3,993,843.000
COVID-19 severity in 2020 (%)	58.157	12.749	13.904	84.702
COVID-19 severity in 2021 (%)	50.691	19.224	12.040	87.040
***Variables of interest***				
Average poverty ($/month)	154.406	116.567	40.210	643.260
Poverty incidence (%)	33.713	23.652	0.130	80.710
Poverty depth (%)	13.236	11.849	0.020	44.950
Poverty severity (%)	7.050	7.356	0.000	29.490
Multidimensional poverty index (%)	32.677	14.052	12.533	75.035
Extreme poverty (%)	35.135	21.546	0.500	77.600
***Control variables***				
Population size (millions)	24.934	35.137	0.098	200.964
Population density (inhabitant/km^2^)	104.883	130.641	3.078	622.962
GDP per capita (current $)	2510.799	2985.829	228.214	16,213.480
Official development assistance (millions; current $)	1024.291	989.438	22.180	4809.970
Health expenditure per capita (current $)	130.805	173.887	18.521	839.773
GINI index (%)	42.515	8.135	27.616	63.026
Corruption control (poor: -2.5; good: 2.5)	-0.639	0.650	-1.716	0.840
Government effectiveness (poor: -2.5; good: 2.5)	-0.794	0.680	-2.478	0.900

*Source*: Authors’ calculations

### Descriptive analyses

**Fig. 1:**
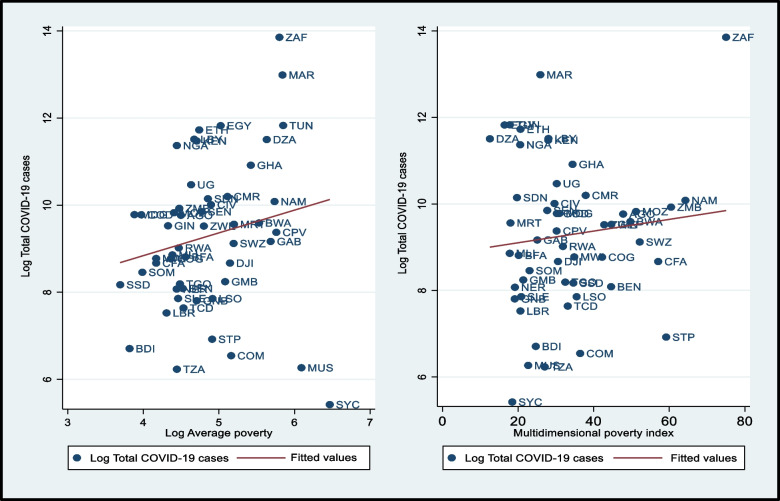
Total COVID-19 cases, average and multidimensional poverty in African countries. Source: Authors’ calculations

The first cases of COVID-19 were reported in most African countries in early March 2020 while the number of confirmed cases increased rapidly after 15 March 2020. As of April 10, 2020, some countries in Africa already had more than 6000 confirmed cases. South Africa had the largest outbreak in Africa with 1,039,161 cases from March to December 2020, while Seychelles had the lowest number of confirmed cases over the same period, estimated at 226 (see Fig. 1). This figure shows a scatter plot of the relationship between the number of COVID-19 cases and average multidimensional poverty in African countries in 2020. However, the number of infections has most likely been underestimated in Africa due to the lack of screening capacity in many countries [34]. Although the number of confirmed cases remains low, and when comparing Africa to other continents of the world, the negative effects are still noticeable, including the contraction of economic activity leading to a drastic decline in household livelihoods, increasing/aggravating the level of poverty in Africa, especially in the south of the Sahara [3, 37].

Furthermore, recall that most economies of African countries are informal, households in these countries tend to face higher levels of informality coupled with lack of social protection [47, 48], and have no income replacement or savings in the event of unexpected external shocks such as COVID-19. This unexpected external shock such as the COVID-19 pandemic is more prone to poverty in the African context. Therefore, social distancing measures to control the virus may be ineffective for African populations, as staying at home and no work implies the loss of revenue crucial to their livelihoods. In this case, we consider average poverty and multidimensional poverty^4^ to link confirmed COVID-19 cases to poverty. Actually, these variables give us useful information on the average poor population by taking into account factors such as education, health, and standard of living of the populations in each African country. These relationships appear to be positive and suggest that higher levels of poverty are associated with higher rates of COVID-19 (see Fig. 1). Moreover, additional evidence is presented in the empirical section.

## Results and discussion

### Basic results

The results presented below highlight the effects of poverty on the spread of COVID-19 in African countries in 2020. Table 2 presents the OLS results when the dependent variable is the total number of cases. While Table 3 presents the results when the dependent variable is the severity of the disease. In both tables, poverty indicators such as average poverty, poverty incidence, poverty depth, poverty severity, multidimensional poverty index and extreme poverty are alternately introduced into models giving six respective specifications. In the case of Table 2, the estimated coefficients of the poverty indicators are positive and significant at the 1% (for poverty incidence and depth of poverty), 5% (average poverty and severity of poverty) and 10% (for extreme poverty and multidimensional poverty) levels. Similarly, in the case of Table 3, the estimated coefficients of the poverty indicators are positive and significant at the 5% (for extreme poverty and depth of poverty) and 10% (for average poverty, incidence of poverty, severity of poverty and the multidimensional poverty index) levels.

**Table 2:** Relationship between COVID-19 spread and poverty in Africa in 2020

	**Log Total COVID-19 cases**					
	**(1)**	**(2)**	**(3)**	**(4)**	**(5)**	**(6)**
Log (Population size)	0.935***	0.877***	0.863***	0.860***	0.868***	0.898***
	(0.225)	(0.144)	(0.164)	(0.175)	(0.224)	(0.188)
Log (Population density)	-0.201	0.139**	0.150	0.160***	0.185**	0.202*
	(0.123)	(0.105)	(0.105)	(0.107)	(0.124)	(0.117)
Log (GDP per capita)	0.579*	0.393*	0.549***	0.620***	0.653**	0.506
	(0.290)	(0.230)	(0.181)	(0.175)	(0.259)	(0.313)
Log (Official development assistance)	0.018	-0.167***	-0.135	0.120	-0.059**	-0.025*
	(0.241)	(0.157)	(0.173)	(0.184)	(0.242)	(0.218)
Log (Health expenditure)	0.148	-0.110*	-0.133***	-0.138**	0.222	-0.116*
	(0.248)	(0.173)	(0.190)	(0.204)	(0.237)	(0.245)
GINI Index	0.030*	0.074***	0.064***	0.057***	-0.032	0.035*
	(0.017)	(0.020)	(0.021)	(0.021)	(0.149)	(0.017)
Corruption control	-0.416***	-0.260	-0.227	-0.244***	-0.303**	-0.456
	(0.701)	(0.526)	(0.550)	(0.567)	(0.692)	(0.671)
Government effectiveness	-0.287*	-0.551**	-0.450	-0.402*	-0.084	-0.184*
	(0.707)	(0.510)	(0.524)	(0.545)	(0.633)	(0.616)
**Average poverty**	0.002**					
	**(0.004)**					
**Poverty incidence**		0.038***				
		**(0.011)**				
**Poverty depth**			0.056***			
			**(0.019)**			
**Poverty severity**				0.074**		
				**(0.029)**		
**Multidimensional poverty index**					0.033*	
					**(0.089)**	
**Extreme poverty**						0.016*
						**(0.009)**
Constant	-11.590***	-10.870***	-11.690***	-11.940***	-9.737	-9.526**
	(3.785)	(2.642)	(3.009)	(3.241)	(6.264)	(3.638)
**R**^**2**^	**0.634**	**0.717**	**0.686**	**0.671**	**0.632**	**0.652**
**Chi2**	**11.200**	**21.860**	**16.730**	**15.140**	**13.190**	**11.250**
**Prob (Chi2)**	**0.000**	**0.000**	**0.000**	**0.000**	**0.000**	**0.000**
**N**	**52**	**52**	**52**	**52**	**52**	**52**

*Note*: Significance *** *p* < 0.01; ** *p* < 0.05; * *p* < 0.1; (.) Standard deviations

**Table 3:** Relationship between COVID-19 severity and poverty in Africa in 2020

	**Log severity of COVID-19**					
	**(1)**	**(2)**	**(3)**	**(4)**	**(5)**	**(6)**
Log (Population size)	0.013***	0.030***	0.028	0.027***	0.032**	0.033*
	(0.033)	(0.028)	(0.029)	(0.030)	(0.031)	(0.028)
Log (Population density)	-0.015	0.008*	0.007***	0.008**	0.022*	0.017
	(0.038)	(0.032)	(0.030)	(0.031)	(0.038)	(0.038)
Log (GDP per capita)	0.163*	0.102*	0.120*	0.134**	0.143*	0.126
	(0.094)	(0.060)	(0.060)	(0.066)	(0.084)	(0.077)
Log (Official development assistance)	-0.018**	-0.023	-0.022	0.021	-0.008**	-0.002*
	(0.042)	(0.038)	(0.038)	(0.037)	(0.036)	(0.034)
Log (Health expenditure)	-0.053	0.002	-0.001***	-0.004***	0.039	-0.009
	(0.044)	(0.042)	(0.044)	(0.045)	(0.037)	(0.047)
GINI Index	0.003	0.012*	0.013*	0.013*	0.031***	0.006
	(0.004)	(0.006)	(0.006)	(0.006)	(0.027)	(0.004)
Corruption control	-0.005***	0.013	-0.002*	-0.004*	0.029	-0.037**
	(0.163)	(0.158)	(0.150)	(0.149)	(0.176)	(0.179)
Government effectiveness	-0.092	-0.217**	-0.222*	-0.221	-0.150***	-0.156*
	(0.161)	(0.151)	(0.148)	(0.148)	(0.157)	(0.159)
**Average poverty**	0.001*					
	**(0.001)**					
**Poverty incidence**		0.006*				
		**(0.003)**				
**Poverty depth**			0.012**			
			**(0.007)**			
**Poverty severity**				0.018*		
				**(0.010)**		
**Multidimensional poverty index**					0.016*	
					**(0.015)**	
**Extreme poverty**						0.002**
						**(0.002)**
Constant	2.222**	2.183**	2.029**	1.952*	1.415**	2.319**
	(1.050)	(0.917)	(0.960)	(1.002)	(1.484)	(1.008)
**R**^**2**^	**0.222**	**0.290**	**0.304**	**0.302**	**0.224**	**0.223**
**Chi2**	**11.680**	**10.880**	**10.950**	**1.040**	**11.410**	**10.970**
**Prob (Chi2)**	**0.000**	**0.000**	**0.000**	**0.000**	**0.000**	**0.000**
**N**	**52**	**52**	**52**	**52**	**52**	**52**

*Note***:** Significance *** *p* < 0.01; ** *p* < 0.05; * *p* < 0.1; (.) Standard deviations

Thus, in Table 2, a one-unit increase in poverty indicators such as average poverty, poverty incidence, poverty depth, poverty severity, multidimensional poverty, and extreme poverty results in an increase of 0.2%; 3.8%; 5.6%; 7.4%; 3.3%; and 1.6%, respectively, in the total number of confirmed cases of COVID-19 in African countries. A high level of poverty is therefore favorable to the spread of COVID-19. However, in the case of Table 3, this increase leads to a 0.1%, 0.6%, 1.2%, 1.8%, 1.6% and 2% increase in disease severity respectively. Indeed, unlike in developed countries, the implementation of containment measures to fight the pandemic is not followed by sufficient follow-up measures in several developing countries, including African countries (e.g., social cash transfers of an amount that would allow the most vulnerable people to remain contained). In most African countries, the average amount of social transfers was too low to increase consumption of the poor’s and lift them out of poverty. As a result, the prevalence of poverty in Africa led to crowded markets, congested streets, and shared sanitation facilities, which can increase the spread of disease [39]. Our finding is consistent with that of [3] who find that the informal sector of a work environment is conducive to the spread of COVID-19. In a recent report, the [36] emphasized on the need to address poverty and vulnerability in Africa during this period of the Covid-19 pandemic. This is important in the struggle against the spread of COVID-19. Poor people with few assets, limited access to credit, informal employment, and low wages were particularly affected by the containment measures introduced during the pandemic. Without any consistent support, these people are likely to ignore the measures and thus contribute to the spread of the disease.

With regard to the control variables, the results are analyzed in two groups. The first group consists of variables that have a significant and positive effect on the spread of the disease and the second group of variables that have a significant and negative effect on the spread of the disease. For the first group, the results show that population size, population density, GDP per capita, and inequality (GINI index) have an overall significant and positive effect on the spread of the disease (Tables 2 and 3). The positive effect of population size and density is in line with previous results. [49] show in the case of India that at the beginning of the pandemic, the population size decreases with the number of cases. However, in the long term, this relationship is increasing. Similarly, [3] show that population density has a positive effect on the spread of the disease. This result is explained by the fact that a large population or density would tend to increase the likelihood of intra-community contagions and non-compliance with social distancing measures. The positive effect of the Gini index on the spread of disease is consistent with the work of [2] and [19] who show that income inequality was the cause of non-compliance with barrier measures. The positive effect of GDP per capita is contrary to the results of [31] and [3] who show that it does not exert a significant effect on the spread of the disease. On the other hand, this result is consistent with [50] who find a positive relationship between economic development and disease spread in China. This positive effect may be explained by the fact that economic development is associated with increased inequality [51, 52] which is a catalyst for the spread of the disease [2, 19].

Concerning the second group, the results reveal that health expenditure, official development assistance, control of corruption, and government effectiveness exert a negative and significant effect on the spread of the disease. In the case of health expenditure capturing health system quality, the negative and significant effect is in contrast to the work of [3] who find no significant effect on the disease spread. On the other hand, the reduction in the spread of the disease can be explained by the fact that health expenditures offer the possibility of improving technical facilities in hospitals and supporting containment policies that limit the spread of the disease [53]. Similarly, the negative and significant effect of official development assistance can be explained by the fact that the transferred resources are used to strengthen disease control measures. The negative and significant effect of corruption and government effectiveness is not surprising. Indeed, in many countries, responses to the COVID-19 crisis occurred immediately after the first cases appeared. This response by the various governments helped to stop the evolution of the pandemic. Similarly, the sound and transparent management of resources allocated to disease containment explains the negative effect of corruption control on the spread of the disease.

### Robustness analysis

In order to validate our main results, we perform two robustness tests. The first test analyzes the effects of poverty on the spread of the disease by focusing on the year 2021 (Tables 4 and 5). While the second test analyzes this relationship by focusing on the year 2022 (January-June) (Table 6). In the case of the second robustness test, only the total number of people infected with the disease is used as the dependent variable, because the severity of the disease is not yet available for the year 2022. The results contained in Tables 4, 5 and 6 are broadly consistent with those obtained in Tables 2 and 3. In conclusion, a high level of poverty in African countries is a primary factor in the spread of COVID-19. This result confirms our empirical findings.

**Table 4:** Relationship between COVID-19 spread and poverty in Africa in 2021

	**Log Total COVID-19 cases**					
	**(1)**	**(2)**	**(3)**	**(4)**	**(5)**	**(6)**
Log (Population size)	0.672***	0.623***	0.622***	0.623***	0.630***	0.616***
	(0.116)	(0.088)	(0.096)	(0.098)	(0.095)	(0.106)
Log (Population density)	0.049***	0.062	0.054	0.052***	0.036**	0.055
	(0.097)	(0.096)	(0.095)	(0.094)	(0.098)	(0.098)
Log (GDP per capita)	0.479	0.498*	0.538**	0.540**	0.541**	0.580**
	(0.300)	(0.254)	(0.244)	(0.248)	(0.245)	(0.272)
Log (Official development assistance)	0.137	-0.188*	0.173	0.170	-0.176*	0.182
	(0.115)	(0.095)	(0.105)	(0.107)	(0.098)	(0.110)
Log (Health expenditure)	-0.435**	-0.497***	-0.518***	-0.523***	-0.568***	-0.557***
	(0.168)	(0.161)	(0.181)	(0.188)	(0.184)	(0.172)
GINI Index	0.042***	0.046***	0.039**	0.037**	0.127	0.035***
	(0.013)	(0.015)	(0.014)	(0.014)	(0.078)	(0.012)
Corruption control	-0.201	-0.289**	-0.282	-0.278***	-0.257	-0.318
	(0.338)	(0.325)	(0.333)	(0.335)	(0.338)	(0.351)
Government effectiveness	-0.472**	0.556	-0.623	0.637	-0.612*	-0.652*
	(0.325)	(0.344)	(0.372)	(0.381)	(0.347)	(0.336)
**Average poverty**	0.001*					
	**(0.002)**					
**Poverty incidence**		0.006**				
		**(0.007)**				
**Poverty depth**			0.001***			
			**(0.013)**			
**Poverty severity**				0.002*		
				**(0.021)**		
**Multidimensional poverty index**					0.053**	
					**(0.045)**	
**Extreme poverty**						0.004**
						**(0.007)**
Constant	-8.195***	-7.851***	-7.916***	-7.888***	-10.270**	-8.378***
	(2.530)	(2.554)	(2.653)	(2.689)	(3.881)	(2.942)
**R**^**2**^	**0.777**	**0.778**	**0.775**	**0.775**	**0.779**	**0.777**
**Chi2**	**33.280**	**34.390**	**33.370**	**33.160**	**34.940**	**35.370**
**Prob (Chi2)**	**0.000**	**0.000**	**0.000**	**0.000**	**0.000**	**0.000**
**N**	**52**	**52**	**52**	**52**	**52**	**52**

*Note***:** Significance *** *p* < 0.01; ** *p* < 0.05; * *p* < 0.1; (.) Standard deviations

**Table 5:** Relationship between COVID-19 severity and poverty in Africa in 2021

	**Log severity of COVID-19**					
	**(1)**	**(2)**	**(3)**	**(4)**	**(5)**	**(6)**
Log (Population size)	0.004**	0.007***	0.006***	0.005**	0.008***	0.005*
	(0.050)	(0.049)	(0.049)	(0.050)	(0.051)	(0.049)
Log (Population density)	0.039*	0.044	0.044**	0.044	0.037**	0.039
	(0.040)	(0.038)	(0.037)	(0.037)	(0.039)	(0.040)
Log (GDP per capita)	0.212*	0.185*	0.195**	0.203**	0.208*	0.218**
	(0.116)	(0.096)	(0.094)	(0.096)	(0.104)	(0.101)
Log (Official development assistance)	-0.037**	-0.030	-0.031*	-0.031***	-0.039	-0.037*
	(0.053)	(0.047)	(0.047)	(0.047)	(0.045)	(0.043)
Log (Health expenditure)	-0.044*	-0.026	-0.024	-0.022*	-0.043	-0.048**
	(0.119)	(0.097)	(0.099)	(0.100)	(0.111)	(0.106)
GINI Index	0.001*	0.005***	0.006	0.006	0.010***	0.000
	(0.007)	(0.008)	(0.008)	(0.007)	(0.048)	(0.006)
Corruption control	-0.102**	-0.102	-0.107	-0.107*	-0.094	-0.106**
	(0.234)	(0.244)	(0.241)	(0.240)	(0.251)	(0.244)
Government effectiveness	-0.122	-0.070**	-0.068*	-0.067	-0.110	-0.117*
	(0.166)	(0.181)	(0.189)	(0.191)	(0.182)	(0.178)
**Average poverty**	0.000*					
	**(0.001)**					
**Poverty incidence**		0.003**				
		**(0.004)**				
**Poverty depth**			0.008**			
			**(0.007)**			
**Poverty severity**				0.010*		
				**(0.011)**		
**Multidimensional poverty index**					0.005***	
					**(0.027)**	
**Extreme poverty**						0.001*
						**(0.003)**
Constant	2.119**	2.133**	2.046*	1.997*	1.870	1.984*
	(1.025)	(1.042)	(1.071)	(1.096)	(1.984)	(1.080)
**R**^**2**^	**0.303**	**0.312**	**0.313**	**0.314**	**0.303**	**0.304**
**Chi2**	**13.490**	**13.920**	**14.110**	**14.280**	**3.340**	**3.620**
**Prob (Chi2)**	**0.000**	**0.000**	**0.000**	**0.000**	**0.000**	**0.000**
**N**	**52**	**52**	**52**	**52**	**52**	**52**

*Note***:** Significance *** *p* < 0.01; ** *p* < 0.05; * *p* < 0.1; (.) Standard deviations

**Table 6:** Relationship between COVID-19 spread and poverty in Africa in 2022

	**Log Total COVID-19 cases**					
	**(1)**	**(2)**	**(3)**	**(4)**	**(5)**	**(6)**
Log (Population size)	0.643***	0.585***	0.585***	0.586***	0.591***	0.576***
	(0.115)	(0.090)	(0.097)	(0.098)	(0.092)	(0.105)
Log (Population density)	0.054	0.065*	0.055**	0.054	0.044	0.061*
	(0.099)	(0.099)	(0.096)	(0.096)	(0.100)	(0.099)
Log (GDP per capita)	0.460	0.507*	0.541**	0.537**	0.532**	0.583**
	(0.311)	(0.261)	(0.254)	(0.260)	(0.253)	(0.276)
Log (Official development assistance)	0.114	-0.165*	0.149	0.146	-0.160*	0.169
	(0.109)	(0.093)	(0.102)	(0.104)	(0.089)	(0.103)
Log (Health expenditure)	-0.435**	-0.522***	-0.546***	-0.553***	-0.580***	-0.583***
	(0.167)	(0.171)	(0.192)	(0.199)	(0.188)	(0.176)
GINI Index	0.038***	0.037**	0.029**	0.028*	0.114	0.029**
	(0.014)	(0.015)	(0.014)	(0.014)	(0.080)	(0.012)
Corruption control	-0.296*	-0.395	-0.382***	-0.379***	-0.369	-0.439*
	(0.327)	(0.321)	(0.326)	(0.327)	(0.328)	(0.342)
Government effectiveness	-0.598*	-0.741**	-0.815**	-0.831**	-0.769**	-0.815**
	(0.306)	(0.338)	(0.364)	(0.373)	(0.336)	(0.323)
**Average poverty**	0.002**					
	**(0.001)**					
**Poverty incidence**		0.003*				
		**(0.007)**				
**Poverty depth**			0.005*			
			**(0.013)**			
**Poverty severity**				0.012**		
				**(0.021)**		
**Multidimensional poverty index**					0.048***	
					**(0.046)**	
**Extreme poverty**						0.006*
						**(0.007)**
Constant	-7.063***	-6.691**	-6.685**	-6.614**	-8.886**	-7.329**
	(2.549)	(2.626)	(2.707)	(2.736)	(3.940)	(3.005)
**R**^**2**^	**0.771**	**0.768**	**0.768**	**0.768**	**0.771**	**0.771**
**Chi2**	**33.980**	**35.260**	**33.420**	**33.210**	**34.290**	**35.59**
**Prob (Chi2)**	**0.000**	**0.000**	**0.000**	**0.000**	**0.000**	**0.000**
**N**	**52**	**52**	**52**	**52**	**52**	**52**

*Note*: Significance *** *p* < 0.01; ** *p* < 0.05; * *p* < 0.1; (.) Standard deviations

## Conclusion

The objective of this study was to analyze the effect of poverty on the spread of COVID-19 in African countries. To do so, it conducts a cross-country analysis and uses OLS for the empirical analysis. The results reveal a positive and significant relationship between poverty and the spread of COVID-19. Sensitivity analyses confirm this result. Furthermore, the results reveal that population size and density, income inequality, and GDP per capita positively affect the spread of COVID-19, while health spending, official development assistance, government effectiveness, and control of corruption have a negative effect. These results suggest that more attention be paid on the poor in African countries in this time of pandemic. They are generally vulnerable, and support programs targeting them need to be put in place quickly. These programs may include food aid, distribution of supplies, health care support, fee waivers, and interest deferrals. In addition, sensitization of these disadvantaged groups on vaccination against COVID-19 to achieve herd immunity is strongly encouraged.

An important limitation of this study is the level of uncertainty around the quality of the available data on the spread of COVID-19 in African countries. However, as it is common in the econometric literature, misreporting of the dependent variable should not bias the estimated coefficients in the linear regression model if the data have some information content as opposed to simple guesses [54, 55]. Misreporting, however, can inflate the variance of the model, leading to less accurate estimates [3, 55].

In addition, this study focuses on non-compliance with work-related social distancing measures as a channel for the virus spread. However, future studies would gain from further exploration of this hypothesis. They might question on whether poorer people have larger households, or whether poorer people spend more time together, so that the spread of the virus is further increased. Future research can therefore take these limitations into account. The debate about COVID-19 and poverty in most developing countries are again ongoing and remains very interesting.

## Data Availability

The data used in this research are available from four sites: the World Health Organization, the World Bank, the World Bank’s PovcalNet report, and Worldwide Governance Indicators.

## Appendix

Table

**Table 7:** Definition of variables

Variables	Definitions	Sources
**Total COVID-19 cases**	The number of confirmed cases of COVID-19 in Africa	[34]
**COVID-19 severity**	Also called the severity of infection, as the extent of illness in people infected with the corona virus	[34]
**Average poverty**	Refers to the average monthly income of people living in a situation of material inferiority compared to the most privileged individuals	[45]
**Poverty incidence**	Measures the poverty rate, i.e., the number of people below the poverty line	
**Poverty depth**	Measures the difference between the average income level of the poor and the poverty line	
**Poverty severity**	Examines income differences within poor populations	
Multidimensional poverty index	Poverty is multidimensional when it is related to several factors, including education, health and standard of living	
**Extreme poverty**	A person is living in extreme poverty if he or she does not have the income to meet basic food needs, usually defined on the basis of minimum caloric requirements	
**Population size**	The size of the population in the African country	[5]
**Population density**	The average number of inhabitants in Africa given per square kilometer	
**GDP per capita**	Measures the level of per capita income in African countries	
**Official development** **assistance**	Provides information on the importance of global governance in the fight against the spread of the disease	
Health expenditure per capita	The quality of the health care system, as represented by the level of per capita health expenditure in African country	
**Gini index**	This index highlights income inequality in the aggregate population level	[45]
**Corruption control**	Indicators of institutional quality (poor: -2.5; good: 2.5)	
Government effectiveness	Indicators of institutional quality (poor: -2.5; good: 2.5)	[46]

*Source*: Authors

*African countries included in this research*: Algeria; Angola; Benin; Botswana; Burkina Faso; Burundi; Cameroon; Cape Verde; Central African Republic; Chad; Comoros; Congo; Cote d’Ivoire; Democratic Republic of Congo; Djibouti; Egypt; Eswatini; Ethiopia; Gabon; Gambia; Ghana; Guinea; Guinea-Bissau; Kenya; Lesotho; Liberia; Libya; Madagascar; Malawi; Mali; Mauritania; Mauritius; Morocco; Mozambique; Namibia; Niger; Nigeria; Rwanda; Sao Tome and Principe; Senegal; Seychelles; Sierra Leone; Somalia; South Africa; South Sudan; Sudan; Tanzania; Togo; Tunisia; Uganda; Zambia; Zimbabwe

7

## Abbreviations

COVID-19: Coronavirus disease 2019
GDP: Gross Domestic Product
IMF: International Monetary Fund
SSA: Sub-Saharan Africa
UNCTAD: United Nations Conference on Trade and Development
UNECA: United Nations Economic Commission for Africa
WAEMU: West African Economic and Monetary Union
WHO: World Health Organization

## Footnotes

1 Lack of clean water, which is crucial to prevent COVID-19 by washing hands. They lack adequate sanitation, which is a major cause of infectious diseases, affecting the immune system and thus compromising the cure of COVID-19. They have inadequate spaces and overcrowded homes and are more exposed to the disease. However, these households cannot maintain a safe physical distance when necessary. Among these households, there may be elderly people who constitute a population at risk. In addition, a high dependency ratio is also associated with greater vulnerability of these households to COVID-19 shock. Finally, poor families often rely on public canteens, and being housebound exposes them to food insecurity, creating difficult conditions to comply with backward measures.

2 These nose plugs are usually traditionally sewn and room, which is not the case with rich people who use imported medical nose plugs.

3 This index is calculated by the [34] considering the period from March 2020 to December 2020.

4 The multidimensional poverty index was calculated by the World Bank, and is published in its PovcalNet report in 2019.
